# From assessment to intervention: evidence-based approaches in tardive dyskinesia

**DOI:** 10.1017/S1092852925000082

**Published:** 2025-02-12

**Authors:** Desiree Matthews

**Affiliations:** Different MHP, PC, Charlotte, NC, USA

**Keywords:** Tardive dyskinesia (TD), VMAT2 inhibitors, early detection, screening, patient-reported outcomes

## Abstract

Tardive dyskinesia (TD) is a potentially irreversible movement disorder induced by dopamine receptor-blocking agents, including antipsychotics. Despite progress in antipsychotic medications, TD remains widely prevalent even in the era of second-generation antipsychotics. Early detection is critical for preventing irreversible damage and minimizing the disorder’s impact on patients’ daily lives. Risk factors for TD include advanced age, female sex, medical comorbidities, and prolonged use of dopamine receptor-blocking agents (DRBAs). Effective screening for TD should incorporate evidence-based screening techniques such as the Abnormal Involuntary Movement Scale (AIMS) and informal methods to capture a comprehensive view of TD’s severity and impact. Combining these approaches allows for a thorough assessment of both healthcare practitioner-perceived severity and patient-reported effects on daily life. Modern treatment options, including vesicular monoamine transporter 2 (VMAT2) inhibitors like valbenazine and deutetrabenazine, have demonstrated significant efficacy and safety in clinical trials. Approved by the FDA in 2017, these medications enable continued psychiatric care while managing TD symptoms. Long-term studies support their sustained efficacy and safety, underscoring the importance of individualized, evidence-based treatment plans to improve patient outcomes.

## Introduction

Tardive dyskinesia (TD) is a potentially irreversible drug-induced movement disorder that is caused by the use of dopamine receptor-blocking agents (DRBAs), which include antipsychotics. The term was first used in medical parlance by Faurbye and others in 1964 for patients who developed chronic involuntary movements after antipsychotic treatment for several months.^1^ Motorically, the TD movements are choreiform or athetoid and the most affected regions are the oral, buccal, lingual areas and the extremities.^2^ In the era of second-generation antipsychotic use, TD unfortunately remains widely prevalent. It is estimated that in 2016 approximately 573,000 individuals were diagnosed with TD with projections of a continued increase in newly diagnosed patients with TD through 2025 due to the anticipated increase in the number of patients treated with antipsychotics.^3^ Approximate prevalence rates among patients taking first-generation antipsychotics (FGAs) and second-generation antipsychotics (SGAs) are 30% and 20%, respectively.^4^ Due to TD’s potentially irreversible nature, it is important to examine screening strategies for earlier diagnosis of TD, identify patient risk factors for developing TD, as well as consider how the dyskinesias of TD may impact a patient’s daily life. Consideration of these areas can assist healthcare professionals in incorporating individualized evidence-based treatment plans for patients with TD including the use of vesicular monoamine transporter 2 (VMAT2) inhibitors.

## Impact of early detection on improving patient outcomes and quality of life

Tardive dyskinesia is a delayed-onset drug-induced movement disorder which may manifest several months or years from the time the patient starts using antipsychotic treatment or other forms of DRBAs. The likelihood of delayed onset calls health care professionals to increase vigilance for TD development in patients undergoing treatment with these agents. If the condition is not identified and addressed promptly, it can often lead to permanent and irreversible movement disorder.^4^ ^,^^5^ Therefore, detection of TD at early stages is essential in mitigating the long-term effects on individuals as well as a potential chance at reversibility in some cases where DRBA treatment can be safely stopped. Understanding risk factors for TD can assist with treatment planning to minimize the risk of TD as well as serve as a guide for when healthcare professionals may need to be at an elevated awareness of the potential development of TD and thus guide our screening and suspicion for possible TD. Factors that increase the risk of TD development include patient-related factors of older age, female sex, co-existing medical comorbidities such as diabetes and HIV, and substance abuse. Treatment-related factors that increase a patient’s risk of developing TD include longer duration and higher doses of DRBAs, history of another drug-induced movement disorder (drug-induced parkinsonism, akathisia, dystonia), and treatment with FGAs.^6^

Early detection and management of TD is critical as recent evidence shows that many patients living with TD experience impact and impairment in their daily lives. A recent survey of outpatients with possible TD reported that 70%–80% were aware of their movements and that 50%–60% felt self-conscious or embarrassed by their movements. Regardless of psychiatric disorder, over 30% of patients reported that their involuntary movements had “some” or “a lot” of impact on their ability to do their usual activities, be productive, and socialize.^7^ Furthermore, results from an online survey of patients living with TD and their caregivers revealed a substantial burden of TD on multiple domains of life including physical, psychological, and social functioning. Among those patients surveyed, 90% of patients reported that TD impacted their physical functioning including falling asleep, exercising, doing household chores, holding onto things (eg, glass or fork), having to eat slowly to avoid choking, having trouble chewing, and speech difficulties interfering with the ability to work. In addition to physical impairments, 75% of patients living with TD reported impact and impairments in socialization including leaving the house, limiting social activities, and enjoying activities they do for fun. Moreover, 80% of patients in this survey living with TD had impacts and impairments on psychological domains including feeling sad, irritable, anxious, and embarrassed. In addition to the impact on the patient living with TD, this survey found that caregivers too were impacted by caring for someone with TD including one-third “often” or “always” feeling anxious or worried because of the patient’s TD.^8^

Increasing research into the experiences of patients living with TD and its effects on both patients and caregivers highlights the importance of detecting TD as early as possible. Early detection is crucial to prevent or reduce the impact and impairment that TD can cause.

## Current landscape of tardive dyskinesia screening

Diagnosing TD combines both physical evaluation and clinical assessment to identify the symptoms and determine their severity. In order to effectively diagnose TD early, routine, and regular screening in all patients taking DRBAs should occur. According to the American Psychiatric Association (APA), for all patients treated with antipsychotics, a clinical assessment of drug-induced movement disorders should be conducted at baseline and every follow-up visit. Furthermore, measurement-based assessments such as the Abnormal Involuntary Movement Scale (AIMS) should be conducted at baseline every 6 months for high-risk patients, and every 12 months for other patients.^9^ This mirrors recommendations made in 2020 from a modified Delphi panel which recommended that a brief, clinical assessment for TD should be performed at every clinical encounter for all patients taking antipsychotics or other DRBAs regardless of the degree of risk for TD.^10^

## Formal measurement based tools for TD

The APA recommends that individuals at risk for TD should be screened with a formal measurement-based tool such as the Abnormal Involuntary Movement Scale (AIMS). AIMS is widely used as the gold standard for screening for potential dyskinesias because of its ability to measure the severity of involuntary movements, monitor changes, and inform the right treatment options.^11^ The AIMS is a 12-item observer-rated scale developed to assess the severity of tardive dyskinesia and follow its progression over time. For the AIMS examination, seven body regions are observed for signs of involuntary movements of TD including muscles of facial expression, lips, perioral area, jaw, tongue upper extremities, lower extremities, and trunk. Movements are rated on a scale from zero meaning “none” or “normal” movement to four indicating “severe” movements. The total AIMS scores are calculated by adding up the scores from items one through seven to produce an AIMS total dyskinesia score. There are additional items rating the global severity of abnormal movements, the patient’s incapacitation and awareness of movement, and dental status.^12^ In one quality improvement study conducted in adult patients taking antipsychotics, it was found that implementation of the AIMS in routine monitoring of TD improved patient outcomes with the detection and treatment of TD.^13^

## Informal tools to identify and assess TD

Along with the AIMS, informal screening tools can be used during clinical assessments and play a key role in the ongoing screening and management of TD symptoms.

The APA guidelines suggest that an informal screening is necessary for every visit for patients who are at risk for developing TD.^8^ ^,^^14^ A potential benefit of informal screening tools is that they can offer open-ended information that paints a real picture of the extent of the effects of TD on the patient and the caregiver.^11^ Combining insights from formal tools like the AIMS with informal assessment approaches can provide a clearer clinical picture of movement severity relative to daily functioning, and provide caregiver and patient perspectives of the impact of dyskinesias, rather than relying solely on the AIMS total dyskinesia score. Citrome et al. also explain that through verbal questioning, a lot can be understood about how TD symptoms are affecting the patients at the initial stages.^15^ A strong and comprehensive result of assessment can be obtained when a formal assessment tool is used hand-in-hand with informal tools, namely unstructured observations, questions, and discussions with the patient and the caregiver. In 2020, a consensus panel of TD experts made recommendations that as part of routine clinical practice assessment of the impact of TD should be made to help guide treatment decisions, with input from the patient, caregiver, and family members. Key domains for assessing overall impact were suggested to include social, physical, vocational, and psychological function and the impact of TD on the underlying mental health disorder.^16^

## IMPACT-TD scale

The IMPACT-TD scale was developed based on recommendations on the importance of assessing the functional impact of TD. The IMPACT-TD scale is an easy-to-use clinical scale to help measure the functional impact of TD in practice settings. The IMPACT-TD scale takes into account the four functional domains of the patient: social, psychological/psychiatric, physical, and vocational aspects. Each dimension receives a value between 0 (no impact) and 3 (severe, significant, and detrimental impact), based on information obtained from patient and caregiver feedback and clinical observation.^17^ This scale can demonstrate the extent to which TD impacts a life outside of what is apparent in a clinical context and may be overlooked in traditional assessments. This can further aid in treatment planning, including when to initiate treatment for TD. Additionally, this tool could be used over time, as the impact of TD may wax and wane, or in instances in which assessment of the resolution or reduction of the impact of impairment is being determined in clinical interviewing.

## MIND-TD questionnaire

The MIND-TD questionnaire was developed to assist healthcare professionals in facilitating a discussion about the risks, symptoms, and impact of TD. The questionnaire was developed in collaboration with expert healthcare professionals and was tested in clinical practice for further revision and refinement. This is another example of an informal tool to screen and assess potential TD. It is recommended to be administered to patients at risk for developing TD or who have a current diagnosis.

The questionnaire contains two parts. The first part is the “MIND” screening section and any trained staff member can administer this in person, via telemedicine, or in an audio-only encounter. Part 1 of the questionnaire includes yes or no questions for each of the following topics: presence of extra or unwanted movements (Movement); feelings of embarrassment of self-consciousness (Impact); whether anyone else noticed the movements (Notice); and if the movements interfere with everyday routines (Daily Activities). Part 2 of the MIND-TD questionnaire includes 9 items (Thorough Interview) that ask patients about physical difficulties such as eating, speaking, walking, and gripping objects in addition to three instructions to determine any presence of speech difficulties that could be indicative of TD in the oral, buccal, lingual region. The second section of part 2 (Differentiate) includes checklists of movements consistent with TD and drug-induced parkinsonism along with an item related to akathisia. Part 2 requires visual observation either in person or conducted via video.^18^

Both the IMPACT-TD scale and MIND-TD questionnaire may provide healthcare professionals with valuable screening tools in assisting with the assessment of TD holistically. Although the AIMS can provide measurement-based care and can help monitor the severity of movements observed by the healthcare professional, it may not fully capture the day-to-day impact and impairment that TD may have on the patient. Even “mild” TD can cause social anxiety, and problems with daily activities including eating, speaking, breathing, and walking.^16^ This subjective experience may not be fully captured in the AIMS alone. These two screeners, in combination with a structured assessment such as the AIMS, may be able to provide a clearer picture to guide the urgency and need for treatment of TD.

## Measurement-based care beyond the AIMS: patient-reported outcomes

In clinical practice, measurement-based care tools such as the Abnormal Involuntary Movement Scale (AIMS) have been adopted due to little else being available. The AIMS, originally developed by the US National Institute of Mental Health has been the gold standard, despite difficulty with implementation in routine clinical practice.^19^ In addition, the use of the AIMS may be problematic if clinicians administering the AIMS do not have a protocol for rating, which could potentially lead to wide variability in ratings within the clinic. It can be a challenge for those with limited experience with the AIMS due to the lack of detailed instructions and descriptors for scoring in the original manuscript. Because the AIMS measures the frequency and amplitude and distribution of movements, using the AIMS score to assess severity alone may not be sufficient.^20^ From a clinical standpoint, relying solely on the total AIMS score to measure TD is insufficient. For instance, a score of 3 in one body region may be considered “mild” according to the AIMS, but it could still significantly impact the patient’s quality of life. Therefore, the total AIMS score alone is not a reliable indicator of the true impact of TD on a patient. In addition, TD can wax and wane during the day and over time—thus presentation in the clinic alone may be a poor indicator of the actual severity or burden of TD.^21^ For clinical practice, a validated, easy-to-use measure for assessing the impact of TD from the patient’s perspective would be a beneficial tool for clinicians.

In 2024, an initial manuscript was published for such a scale called the Tardive Dyskinesia Impact Scale (TDIS), a novel validated patient-reported outcome measure. The TDIS is an 11-item questionnaire developed to understand how TD affects current daily functioning over the previous 7 days, which is scored on a 5-point Likert scale ranging from 0 (no impact) to 4 (most impact). TDIS scores can range from 0-44, with higher scores representing greater impact. The TDIS was adapted from the Tardive Dyskinesia Rating Scale (TDRS) and qualitative and quantitative research was used to develop the scale. In addition, the TDIS was developed in conjunction with patients with TD and caregivers to assist in developing questions relevant to the patient’s experience with TD and free of medical language. Results from psychometric testing indicate that the TDIS can capture the severity of the effects of TD from the patient’s perspective and capture key patient experiences including the physical, social, and emotional impacts of TD.^11^ Having a validated measure on patient-reported outcomes of the impact of TD would serve as a valuable tool for healthcare providers to guide treatment decisions and assist with monitoring TD impact over time and could complement a clinician-rated scale, such as the AIMS in a holistic and comprehensive assessment of TD.

## Modern evidence-based strategies for management and treatment of tardive dyskinesia

Up until recently, there have been few options to manage TD, none of which were approved by the Food and Drug Administration (FDA). Even though TD is quite common, affecting 20%-50% of patients who take antipsychotic medications, the previous decades-long absence of an effective treatment has led to a sense of therapeutic pessimism.^22^ ^,^^19^ Historically, there were minimal options to offer individuals living with TD prior to 2017 and no medication intervention carried FDA approval. In 2013, the American Academy of Neurology (AAN) cited limited evidence for the treatment of TD—noting clonazepam and ginkgo biloba as level B evidence and tetrabenazine and amantadine as level C evidence. Management of TD was approached in several “off label” ways such as attempting to switch a first-generation antipsychotic to a second-generation antipsychotic with “perceived” less risk of TD, but there is little evidence to support this.^23^

In cases where DRBAs can be safely stopped such as in individuals with non-psychotic disorders, reversability, and remission may occur. In a retrospective study of patients with TD in non-psychotic conditions where the DRBA could be discontinued, 13% of patients experienced symptom resolution.^24^ In many cases medications such as antipsychotics, are the mainstay of treatment for individuals with psychotic disorders and cessation is not an option, leaving healthcare professionals and patients previously having to endure living with TD without treatment interventions with robust efficacy.

Modern approaches to treating tardive dyskinesia (TD) now allow patients to continue their stable psychiatric medications, DRBAs, such as first or second-generation antipsychotics. This means patients do not have to stop treating their mental health condition to manage TD symptoms. Valbenazine and deutetrabenazine, vesicular monoamine transporter 2 (VMAT2) inhibitors, were approved by the FDA in 2017 for the treatment of adults with TD. Both VMAT2 inhibitors are indicated for adults with TD and can be added to most stable mental health and medical medications. With FDA approval of the two VMAT2 inhibitors, both the APA and AAN have updated their treatment guidelines for the management of adults with TD to include first-line treatment for TD with VMAT2 inhibitors as level A evidence.^25^ Furthermore, the APA guidelines suggest that healthcare professionals consider VMAT2 inhibitors for moderate to severe cases of TD and where there is evidence of significant impairment of psychosocial functioning, physical health, socialization domains, and vocational activities.^9^ This emphasizes the importance of a holistic approach in the assessment and management of TD to be based on not only the severity of movements as measured formally by AIMS but also tailoring treatment based on the perceived impact and impairment of the patient, family, and caregivers affected by TD.

Both VMAT2 inhibitors were evaluated in short-term, double-blind placebo-controlled clinical trials of adults with TD and underlying mental health disorders including psychotic disorders and mood disorders. In short-term trials, both VMAT2 inhibitors showed robust efficacy in comparison to placebo at reducing involuntary movements of TD, with higher doses conveying greater efficacy. Valbenazine and deutetrabenazine were also generally well tolerated in the short-term placebo-controlled trials.^26^

## Long-term safety and efficacy data for deutetrabenazine

Long-term safety data for deutetrabenazine remains encouraging. Deutetrabenazine was studied in a 3-year open-label extension study of 343 patients who rolled into the study from the previous short-term clinical trials of deutetrabenazine. Following a washout phase, patients underwent a titration period for 6 weeks and followed over 3 years. It is important to note that patients were titrated until adequate dyskinesia control was achieved or a clinically significant adverse event occurred or the maximum dose of 48 mg/day was reached (36 mg/day for patients receiving a strong CY2D6 inhibitor). In this study, treatment was well tolerated and no new safety signals were identified. The most common adverse events that occurred included anxiety, somnolence, depression (excluding self-injury/suicide), weight decrease, and urinary tract infections. By the end of the study period, the average dose of deutetrabenazine was 39.4 mg at week 145. Treatment in this open-label study showed robust and sustained reductions in dyskinesia with two-thirds of patients achieving a 50% or more reduction from baseline and approximately 40% of patients achieving a 70% or more improvement in total AIMS score from baseline. Patient-reported outcomes suggest that long-term deutetrabenazine treatment was associated with improved quality of life.^27^ In addition to the initial formulation of deutetrabenazine which is indicated to be taken twice daily with meals, a new formulation of deutetrabenazine was announced in February 2023. The new extended-release formulation is intended to be taken once daily with or without food and may hold promise to simplify dosing for patients and caregivers while retaining efficacy seen in the short- and long-term clinical trials.^28^

## Long-term safety and efficacy data for valbenazine

In addition to short-term clinical trials for adults with TD in KINECT 2 and KINECT 3, valbenazine has been evaluated for long-term safety and efficacy. Those adults in the short-term, 6-week, double-blind, placebo-controlled period of KINECT 3 were eligible to enter a 42-week valbenazine extension period followed by a 4-week washout period in which patients discontinued their valbenazine dose of 40 mg or 80 mg once daily. In the long-term extension period, valbenazine was found to be generally well tolerated and similar to the short-term, 6-week, double-blind, placebo-controlled safety results which included side effects of somnolence, akathisia, and dry mouth. In addition to safety, patients in the long-term extension period in KINECT 3 showed sustained reductions in TD severity over the treatment period of 42 weeks. During the 4-week washout period, TD movements returned back to baseline, suggesting that valbenazine treatment may need to be continued, as clinically appropriate, to maintain reductions in dyskinesias.^29^

In a post hoc analysis of the KINECT 3 extension study looking at remission of TD (defined as AIMS score of 1 or less on all items) during treatment with valbenazine through the total 48 weeks showed 18% of participants on the 40mg dose and 37% of participants on the 80mg dose achieving remission.^30^

Additionally, the KINECT 4 study was conducted, which was a phase 3 open-label study to further evaluate the long-term safety and tolerability of valbenazine 40 mg or 80 mg once daily as well as the long-term efficacy of treatment. The study looked at psychiatrically stable adults with a TD diagnosis (73% of participants had a diagnosis of schizophrenia or schizoaffective disorder and 27% had a mood disorder). 167 participants entered the study and 103 (62%) completed the 48-week treatment and 4-week washout period. The long-term safety profile of valbenazine in KINECT 4 was similar to previous studies; the majority of treatment-emergent adverse events (TEAEs) were mild or moderate and few led to premature discontinuation. A large majority of participants who completed the 48-week treatment had substantial and sustained TD improvements with valbenazine. After 48 weeks of valbenazine, 86% of participants met the threshold of 50% of greater improvement threshold for AIMS reduction; 88%–92% achieved ratings as “much improved” or “very much improved” on the Global Impression of Change-TD and Patient Global Impression of Change. For participants who did not immediately reach the response threshold, many did so with sustained treatment. Similar to the previous KINECT 3 study, once valbenazine was stopped, dyskinetic movements returned to baseline severity.^31^

## Conclusion

Tardive dyskinesia (TD) remains a significant concern for individuals treated with dopamine receptor-blocking agents (DRBAs), especially antipsychotics. Despite the potential for irreversible motor disturbances, early detection, screening, and evidence-based interventions may substantially mitigate the long-term impacts of TD on patients’ lives. Regular use of formal assessment tools like the Abnormal Involuntary Movement Scale (AIMS), alongside patient and caregiver input through tools like the IMPACT-TD and MIND-TD questionnaires, is essential for comprehensive diagnosis and management. The potential for a validated patient-reported outcome measure, the TDIS, may also fill the gap of having a validated instrument to assess the impact and impairment of individuals living with TD.

With the advent of vesicular monoamine transporter 2 (VMAT2) inhibitors—valbenazine and deutetrabenazine—there are now FDA-approved and effective treatment options for TD. These medications, supported by robust clinical data, show significant efficacy in reducing involuntary movements while allowing patients to continue their psychiatric medications. Long-term studies confirm their safety and sustained effectiveness, offering renewed hope for patients affected by TD. As research continues, a holistic, patient-centered approach combining clinical assessments, patient-reported outcomes, and individualized treatment plans remains paramount in improving outcomes for patients with TD.
