# From Assessment to Intervention: Evidence-Based Approaches in Tardive Dyskinesia – ADDENDUM

**DOI:** 10.1017/S1092852925100254

**Published:** 2025-07-25

**Authors:** Desiree Matthews

*This CME activity is provided by HMP Education and Neuroscience Education Institute (NEI).*

## CME/CE Information

**Target Audience:** This activity has been developed for the healthcare team or individual prescriber specializing in mental health. All other healthcare team members interested in psychopharmacology are welcome for advanced study.

**Learning Objectives:** After completing this educational activity, you should be better able to:

- Implement evidence-based screening and diagnostic techniques for the identification of tardive dyskinesia in patients who are at risk
- Apply evidence-based tools and strategies to evaluate the severity and impact of tardive dyskinesia symptoms on patients’ daily lives, including both healthcare practitioner-perceived severity of tardive dyskinesia symptoms and patient-reported impact of tardive dyskinesia on daily life
- Incorporate individualized, evidence-based treatment management plans for patients with tardive dyskinesia

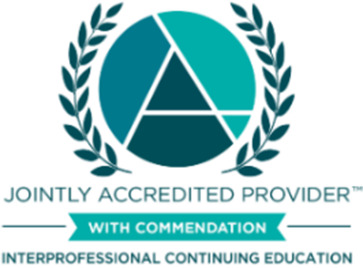
 **Accreditation:** In support of improving patient care, this activity has been planned and implemented by HMP Education and Neuroscience Education Institute (NEI). HMP Education is jointly accredited by the Accreditation Council for Continuing Medical Education (ACCME), the Accreditation Council for Pharmacy Education (ACPE), and the American Nurses Credentialing Center (ANCC), to provide continuing education for the healthcare team.

**Activity Overview:** This activity is best supported via a computer or device with current versions of the following browsers: Mozilla Firefox, Google Chrome, or Safari. A PDF reader is required for print publications. A post-test score of 70% or higher is required to receive CME/CE credit.

**Estimated Time to Complete:** 1 hour.

**Released:** February 12, 2025* **Expiration:** February 11, 2028

*NEI maintains a record of participation for six (6) years.

**Credit Designations:** The following are being offered for this activity:

- **Physician: ACCME *AMA PRA Category 1 Credits*™**
  - HMP Education designates this enduring material for a maximum of 1.00 *AMA PRA Category 1 Credit*™. Physicians should claim only the credit commensurate with the extent of their participation in the activity.
- **Nurse: ANCC contact hours**
  - This continuing nursing education activity awards 1.00 contact hour. Provider approved by the California Board of Registered Nursing, Provider #18006 for 1.00 contact hour.
- **Nurse Practitioner: ACCME *AMA PRA Category 1 Credit*™**
  - American Academy of Nurse Practitioners National Certification Program accepts *AMA PRA Category 1 Credits*™ from organizations accredited by the ACCME.
  - The content in this activity pertaining to pharmacology is worth 1.00 continuing education hour of pharmacotherapeutics.
- **Pharmacy: ACPE application-based contact hours**
  - This internet enduring, knowledge-based activity has been approved for a maximum of 1.00 contact hour (.10 CEU).
  - *The official record of credit will be in the CPE Monitor system. Following ACPE Policy, NEI and HMP Education must transmit your claim to CPE Monitor within 60 days from the date you complete this CPE activity and are unable to report your claimed credit after this 60-day period. Ensure your profile includes your DOB and NABP ID.*
- **Physician Associate/Assistant: AAPA Category 1 CME credits**

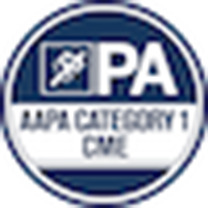
 HMP Education has been authorized by the American Academy of PAs (AAPA) to award AAPA Category 1 CME credits for activities planned in accordance with the AAPA CME Criteria. This internet enduring activity is designated for 1.00 AAPA Category 1 credit. Approval is valid until February 11, 2028. PAs should only claim credit commensurate with the extent of their participation.
- **Psychology: APA CE credits**

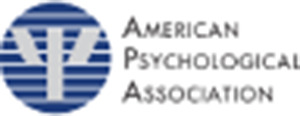
 Continuing Education (CE) credits for psychologists are provided through the co-sponsorship of the American Psychological Association (APA) Office of Continuing Education in Psychology (CEP). The APA CEP Office maintains responsibility for the content of the programs. This activity awards 1.00 CE Credit.
- **Social Work: ASWB-ACE CE credits**
  - As a Jointly Accredited Organization, HMP Education is approved to offer social work continuing education by the Association of Social Work Boards (ASWB) Approved Continuing Education (ACE) program. Organizations, not individual courses, are approved under this program. Regulatory boards are the final authority on courses accepted for continuing education credit. Social workers completing this internet enduring course receive 1.00 general continuing education credit.
- **Non-Physician Member of the Healthcare Team: Certificate of Participation**
  - HMP Education awards hours of participation (consistent with the designated number of *AMA PRA Category 1 Credit*™) to a participant who successfully completes this educational activity.

**Peer Review:** The content was peer-reviewed by an MD, MPH specializing in forensics, psychosis, schizophrenia, mood disorders, anxiety, and cognitive disorders — to ensure the scientific accuracy and medical relevance of information presented and its independence from commercial bias. NEI and HMP Education takes responsibility for the content, quality, and scientific integrity of this CME/CE activity.

**Disclosures:** All individuals in a position to influence or control content are required to disclose any relevant financial relationships. Any relevant financial relationships were mitigated prior to the activity being planned, developed, or presented.

***Faculty Author / Presenter***

**Desiree M. Matthews, MSN, PMHNP-BC**

*Founder and Clinical Director, Different MHP, PC, Charlotte, NC*

Consultant/Advisor: AbbVie, Alkermes, Biogen, Boehringer Ingelheim, Bristol Myers Squibb, Indivior, Janssen Pharmaceuticals, Johnson & Johnson, Neurocrine Biosciences, Sage Therapeutics, Teva Pharmaceuticals

Speakers Bureau: AbbVie, Axsome, Bristol Myers Squibb, Johnson & Johnson, Lundbeck, Neurocrine Biosciences, Otsuka Pharmaceuticals, Teva Pharmaceuticals

The remaining Planning Committee members, Content Editors, Peer Reviewer, and NEI planners/staff have no financial relationships to disclose. NEI and HMP Education planners and staff include Gabriela Alarcón, PhD, Caroline O’Brien, MS, Ali Holladay, Andrea Zimmerman, EdD, CHCP, Brielle Calleo, and Steven S. Simring, MD, MPH.

**Disclosure of Off-Label Use:** This educational activity may include discussion of unlabeled and/or investigational uses of agents that are not currently labeled for such use by the FDA. Please consult the product prescribing information for full disclosure of labeled uses.

**Cultural Linguistic Competency and Implicit Bias:** A variety of resources addressing cultural and linguistic competencies and strategies for understanding and reducing implicit bias can be found in this handout—download me.

**Accessibility Statement**

For questions regarding this educational activity, or to cancel your account, please email customerservice@neiglobal.com.

**Support:** This activity is supported by an unrestricted educational grant from Neurocrine Biosciences.

## Optional Posttest and CME Certificate

*CME credit expires: February 11, 2028*

### Posttest Study Guide

**The posttest can only be submitted online.** The below posttest questions have been provided solely as a study tool to prepare for your online submission.

1. According to the American Psychiatric Association (APA) guidelines, how often should patients with schizophrenia be assessed for tardive dyskinesia using a structured instrument like the Abnormal Involuntary Movement Scale (AIMS)? Age
  - Every 3 months for all patients
  - Every 6 months for high-risk patients and every 12 months for other patients
  - Every 12 months for all patients
  - Only when symptoms are first noticed
2. Which of the following statements about tardive dyskinesia screening tools is correct?
  - The AIMS (Abnormal Involuntary Movement Scale) directly assesses patient-reported impacts of tardive dyskinesia on daily functioning
  - The TDIS (Tardive Dyskinesia Impact Scale) is a clinician-rated scale that focuses on the severity of involuntary movements
  - The IMPACT-TD scale categorizes tardive dyskinesia symptoms into multiple functional domains including social, psychological, physical, and vocational impacts
  - The MIND-TD Questionnaire is designed to be completed solely by caregivers to assess tardive dyskinesia impacts
3. A 39-year-old woman with bipolar II disorder has been using a second-generation antipsychotic for the last year and has developed tardive dyskinesia symptoms rated as mild on the AIMS. She rates the impairment to her social and vocational life as severe on the Tardive Dyskinesia Impact Scale. Based on this information, is treatment with a vesicular monoamine transporter 2 (VMAT2) inhibitor warranted for this individual?
  - No
  - Yes

### Instructions for Optional Posttest and CME Credit

1. Read the article
2. Successfully complete the posttest at https://nei.global/CNS-TD-01
3. Print your certificate

Questions? Email customerservice@neiglobal.com.
